# The Extracellular Domain of Neurotrophin Receptor p75 as a Candidate Biomarker for Amyotrophic Lateral Sclerosis

**DOI:** 10.1371/journal.pone.0087398

**Published:** 2014-01-27

**Authors:** Stephanie R. Shepheard, Tim Chataway, David W. Schultz, Robert A. Rush, Mary-Louise Rogers

**Affiliations:** 1 Department of Human Physiology and Centre for Neuroscience, Flinders University, Adelaide, South Australia, Australia; 2 Neurology Department and MND Clinic, Flinders Medical Centre, Bedford Park, South Australia, Australia; National University of Singapore, Singapore

## Abstract

Objective biomarkers for amyotrophic lateral sclerosis would facilitate the discovery of new treatments. The common neurotrophin receptor p75 is up regulated and the extracellular domain cleaved from injured neurons and peripheral glia in amyotrophic lateral sclerosis. We have tested the hypothesis that urinary levels of extracellular neurotrophin receptor p75 serve as a biomarker for both human motor amyotrophic lateral sclerosis and the SOD1^G93A^ mouse model of the disease. The extracellular domain of neurotrophin receptor p75 was identified in the urine of amyotrophic lateral sclerosis patients by an immuno-precipitation/western blot procedure and confirmed by mass spectrometry. An ELISA was established to measure urinary extracellular neurotrophin receptor p75. The mean value for urinary extracellular neurotrophin receptor p75 from 28 amyotrophic lateral sclerosis patients measured by ELISA was 7.9±0.5 ng/mg creatinine and this was significantly higher (p<0.001) than 12 controls (2.6±0.2 ng/mg creatinine) and 19 patients with other neurological disease (Parkinson's disease and Multiple Sclerosis; 4.1±0.2 ng/mg creatinine). Pilot data of disease progression rates in 14 MND patients indicates that p75NTR^ECD^ levels were significantly higher (p = 0.0041) in 7 rapidly progressing patients as compared to 7 with slowly progressing disease. Extracellular neurotrophin receptor p75 was also readily detected in SOD1^G93A^ mice by immuno-precipitation/western blot before the onset of clinical symptoms. These findings indicate a significant relation between urinary extracellular neurotrophin receptor p75 levels and disease progression and suggests that it may be a useful marker of disease activity and progression in amyotrophic lateral sclerosis.

## Introduction

Amyotrophic lateral sclerosis (ALS) or Motor neuron disease (MND) is a disease characterised by progressive, debilitating paralysis from loss of motor neurons in the cerebral cortex, brain stem and spinal cord and is almost always fatal. The average life expectancy following diagnosis is just 36 months with, most often, patients succumbing to the disease because of denervation of muscles involved in respiration. There is only one therapeutic option for patients, a glutamate antagonist, riluzole, which offers a modest 3–6 month extension in survival [1].

At present there is an urgent need for marker(s) able to measure disease progression for objective monitoring of therapies for human clinical trials and pre-clinical SOD1^G93A^ mice trials [2]. The only validated markers for disease progression are a subjective measure of disability and breathing, called the revised ALS functional rating scale (ALSFRS-r) with a scale from 0 to 48, and time to death [3]. The ALSFRS-r, although the most powerful marker at present is neither objective nor sensitive in the short term, given that individuals are so variable in the course of their disease. The search for more objective biomarkers has encompassed protein based, neurophysiological, and neuroimaging biomarkers, but none have progressed to clinic [4], [5]. Biomarkers that reflect disease progression objectively in both human and mice will improve the analysis of clinical trials and allow for more rapid screening of potential new treatments with fewer patients than required for standard placebo-controlled trials.

Identifying ALS earlier in those with pre-disposing genetic mutations may improve the effectiveness of the only available treatment, riluzole. The reported 6–12 month delay in ALS diagnosis [6] also indicates the necessity for diagnostic and prognostic biomarkers. Early intervention is hindered by an often-lengthy diagnostic process based predominately on clinical assessment and electrophysiological findings [7].

Clinical utility of objective biomarkers may require a combination of biochemical, physiological and imaging based methodologies [2]. There have been a number of searches for CSF biomarkers. CSF biomarker candidates can be classified according to ALS pathological mechanisms such as inflammatory markers, markers of glial response, axonal damage and apoptosis [5]. Neurofilament proteins associated with axon degradation have shown promise. Neurofilament phosphorylated heavy chain (pNF-H) [8] and light chain (NF-L) in the CSF [9] have been investigated as potential biomarkers of ALS. However, the most promising candidates so far appear to be combinations of CSF proteins that improve sensitivity specificity and overall accuracy for diagnosing ALS. These include a combination of neurofilament phosphorylated heavy chain (pNF-H) with complement C3 [8] and a three protein combination including cystatin C and fragments of VEGF [10]. However, none of these biomarkers have been validated in larger independent studies. Although CSF biomarkers show promise the invasive nature of CSF sampling is problematic for ALS patients and especially healthy controls [2]. Therefore searches in other bio-fluids such as urine should not be dismissed and could added to potential panels in large studies.

The common neurotrophin receptor p75 (p75NTR) is highly expressed in motor neurons during embryonic development, down-regulated post-natally and re-expressed during injury [11], [12]. The extracellular domain of p75NTR (p75NTR^ECD^) that is cleaved following pro-apoptotic ligand binding (e.g. nerve growth factor, [13]) has been found in newborn infant urine but declines to low but detectable levels in adult urine even to old age [14]. Importantly, p75NTR^ECD^ was found in urine of rats following sciatic nerve injury but is barely detectable in normal adult rat urine [15]. This indicates that the extracellular portion of the receptor is shed after injury and is excreted in urine. Significantly, p75NTR has been identified histologically in motor neurons of ALS patients post-mortem [16] and the SOD1^G93A^ mouse model of ALS [17]. Our hypothesis is that the appearance of p75NTR^ECD^ in urine has potential as a marker for ALS and possibly disease progression. The objectives of the present study were to determine if urinary p75NTR^ECD^ levels differ between patients and mice with ALS and healthy controls. In addition, pilot studies will determine if p75NTR^ECD^ has potential as a marker of ALS disease progression.

## Materials and Methods

### Study populations

Informed written consent according to the declaration of Helsinki [18] was obtained from all participants and approval to undertake the current study was permitted by the Flinders University of South Australia Southern Adelaide Clinical Human Research Ethics Committee. All participants provided written consent to undertake this current study and the written consent procedure used in this study was approved by The Flinders University of South Australia Southern Adelaide Clinical Human Research Ethics Committee. Urine was collected by the Department of Neurology from patients with ALS according to the revised El Escorial criteria [19] and all other variants were excluded. Patients presented with sporadic (non genetic) ALS as either limb onset or bulbar onset. All patients were also enrolled in the Australian Motor Neuron Disease Registry (AMNDR) where disease progress was recorded including the revised ALS function rating scale (ALSFRS-r [3]). The control group consisted of healthy persons assessed as having no neurological conditions or other illness. We also enrolled people with other neurological diseases including Parkinson's Disease (PD) and Multiple Sclerosis (MS). Patients were diagnosed with PD using the UK Parkinson's Disease Society Brain Bank [20] criteria and those with MS using MRI under the revised McDonald diagnostic criteria [21]. Patients were scored by a neurologist (DWS), blinded to results of the urinary biomarker. Urine samples were collected and stored according to Urine and Kidney Proteome Project Standards [22]. Samples were labelled to ensure anonymity and stored at −80°C in small aliquots until analysis. Protein estimation was performed using Bio-Rad DC Protein assay kit (Hercules, CA). Creatinine was measured by enzymatic analysis using a Roche/Hitachi Modular Analyser.

### Animal Experiments

Approval to undertake experiments using mice with the human SOD1^G93A^ mutant transgene and C57BL/6J mice described in this current study was given by the Flinders University Animal Welfare Committee. Mice with the human SOD1^G93A^ mutant transgene were used as an animal model of ALS and bred and maintained as prescribed by Jax Laboratories [23] in accordance with Flinders University Animal Welfare Committee guidelines. Disease progression in SOD1^G93A^ mice was measured using the hanging wire (grip duration) test [24]. Urine was collected using metabolic cages or from the bladder after euthanasia as previously described [15] from SOD1^G93A^ mice and C57BL/6J healthy controls at 40, 60, 80, 100, 120 days and end-stage disease (145–150 days old). Urinary protein and creatinine measurements were performed as for human urine.

### Detection of p75NTR

p75NTR^ECD^ was identified in urine by immuno-precipitation (IP) using an in-house monoclonal antibody (MLR2 [25]) and western blot (WB) with a goat anti-p75NTR (Sigma-Aldrich). Specificity of the MLR antibodies for p75NTR has been described by our laboratory [25] and elsewhere (e.g [26]). Using MLR 2 as immuno-precipitating antibody, but replacing the detecting goat anti-p75NTR with a rabbit anti-human p75NTR^ECD^ (Alomone Labs) also enabled visualisation of p75NTR bands (See Figure S1). However, the rabbit anti-human p75NTR^ECD^ does not detect mouse p75NTR, so we used a goat anti-p75NTR that detects both human and mouse p75NTR in this study. Briefly, samples were concentrated and washed with PBS using Amicon Ultra 3 kDa centrifugal filters (Merck-Millipore). 500 µg (human urine or cell lysates) or 110 µg (mouse urine) of protein was immuno-precipitated with anti-p75NTR MLR2/Protein G Agarose beads (Merck-Millipore). Samples were then subject to 1D gel electrophoresis and western blot [27] using goat anti-p75NTR as detection. Bovine anti-goat IgG-HRP (Jackson ImmunoResearch Labs) was used as secondary antibody with minimal cross-reactivity to heavy chain IgGs from other animals but detects 25 kDa light chain-IgG. Lysates of cell lines expressing p75NTR (human A875 [28] and mouse NSC34 [25]) were used as positive controls for IP/WB and a fibroblast cell line (BSR) lacking p75NTR [25] was a negative control.

Mass spectrometry was used to confirm the presence of p75NTR^ECD^ in human urine. p75NTR^ECD^ was immuno-precipitated with anti-p75NTR MLR2 as described above and digested overnight at 37°C with glutamyl endopeptidase (Sigma-Aldrich) after reduction with 50 mM DTT and alkylation with 100 mM Iodoacetamide in ammonium bicarbonate (pH 8.0). Peptides were analysed using a LTQ Thermo Orbitrap XL linear ion trap (IT) mass spectrometer fitted with a nanospray source (Thermo Electron Corporation) as described previously [27]. The spectra were searched with Proteome Discoverer 1.3 (Thermo) against the Swissprot database.

### p75NTR^ECD^ measurement

A sandwich ELISA was developed to quantify p75NTR^ECD^ in urine. ELISA plates (96-well plates, Costar Corning) were coated for 18 h with anti-p75NTR^ECD^ MLR1 [25] (4 ug/ml in 25 mM sodium carbonate 25 mM sodium hydrogen carbonate, 0.01% thimerosol, pH 9.6) at 4°C. Wells were blocked with sample buffer containing PBS, 2% BSA and 0.01% thimerosol, pH 7.4, for 1 h at 37°C. Recombinant human p75NTR^ECD^ (aa:29–250, R&D systems) or recombinant mouse p75NTR^ECD^ (aa:20–243, R&D systems) and samples were diluted in sample buffer and incubated for 20 h at RT. Goat anti-p75NTR^ECD^ (R&D systems) was used as detection for 1 h RT and then secondary antibody (bovine anti Goat IgG-HRP; Jackson ImmunoResearch) for 1 h at RT. The peroxidase reaction was developed using TMB (BioRad) and stopped with 2 M sulphuric acid. Between steps, plates were washed 4-times with wash buffer (PBS, 0.05% Tween20, 0.01% thimerosol, pH 7.4). A commercial sandwich ELISA for human p75NTR^ECD^ was also used (R&D systems). Plates were read at 450 nm with a PerkinElmer Victor-x4 Plate Reader (Waltham, MA) and results analysed by one-way ANOVA, with Bonferroni's multiple comparison test using Prism6 and alpha significance level of 0.01% (La Jolla, USA). The diagnostic ability of p75NTR^ECD^ was tested by Receiver Operating Characteristic (ROC) curves (Prism6) and the cut-off levels for diagnosis using the Youden Index [29]. Data comparisons between two groups were performed using the Mann–Whitney test for two independent groups, using Prism6 and alpha significance level of 0.01%.

## Results

### Detection of p75NTR^ECD^ in ALS Patients and SOD1^G93A^ Mice

**Figure 1:**
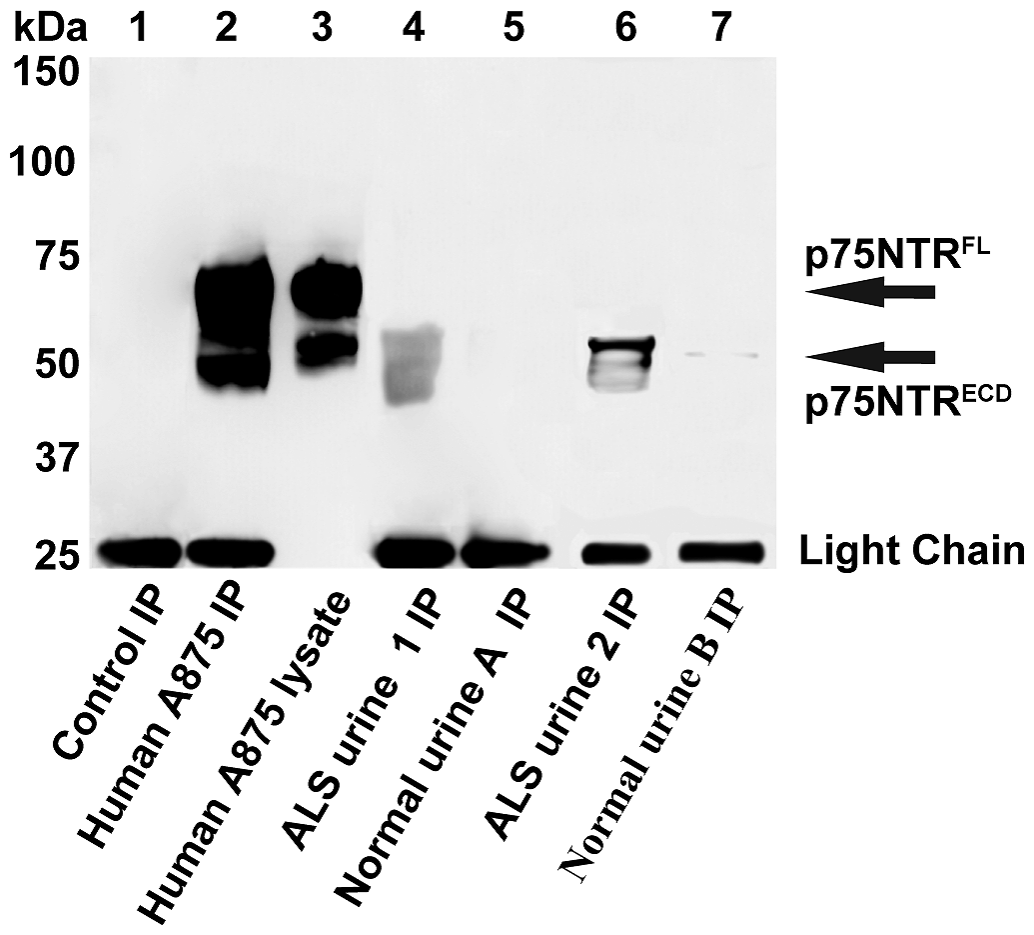
Human p75NTR extracellular domain (p75NTR^ECD^) was detected in urine of humans living with ALS by immuno-precipitation/western blot. Human p75NTR^ECD^ was detected after immuno-precipitation (IP) of 500 µg urinary protein from ALS patients 1 and 2 (lane 4, lane 6) but not from healthy controls A and B (lane 5, lane 7). Human p75NTR^ECD^ (∼50–55 kDA) and full-length p75NTR (p75NTR^FL^, ∼70–75 kDA) from A875 melanoma cells (5 µg lane 3) was enriched by IP of 500 µg of cell lysate (lane 2). No p75NRTR was detected after IP of control cells lacking p75NTR (BSR, 500 µg, lane 1). The light chain of IgG (25 kDa band) was detected after western blot (WB) of samples subject to IP.

p75NTR^ECD^ was detected in human urine of ALS patients by a combined IP/WB procedure using MLR2 as capture and a goat anti-p75NTR^ECD^ as detection (Fig. 1). The specificity of the procedure is indicated by the detection of full-length p75NTR (70–75 kDA, p75NTR^FL^) and p75NTR^ECD^ (50–55 kDA) detected in human A875 cells (lane 3) and enriched by IP/WB (lane 2). There were no protein bands found when control cell lysates lacking p75NTR were subject to IP (lane 1). The specificity of the IP procedure is further demonstrated by the extensive validation of the MLR2 capture antibody [25], [26], [30]. In addition, using MLR2 as capture antibody but replacing the goat anti-p75NTR^ECD^ detecting antibody (used in Fig.1) with a rabbit anti-p75NTR^ECD^ (Figure S1) indicates MLR2 is specific for p75NTR. Both Fig. 1 (lane 3 and 2) and Figure S1 (lane 1 and 2) show p75NTR^FL^ (70–75 kDA) and p75NTR^ECD^ (50–55 kDA) is immuno-precipitated from A875 cell lysates using MLR2. In Fig. 1 the immuno-precipitated A875 protein band for p75NTR^ECD^ (lane 2) appears at a slightly lower molecular weight compared to that in the lysate (lane 3). This is most likely reflective of the detecting goat anti-p75NTR^ECD^ being very sensitive for p75NTR^FL^ and p75NTR^ECD^ so that the bands appear to merge into large broad bands.

The protein bands shown from the IP of 2 separate ALS patient's urine (Fig. 1. lane 4, lane 6) on the WB indicates broad p75NTR^ECD^ reactivity in urine, all between 40 and 55 kDA. No (lane 5) or very faint (lane 7) protein bands were detected in normal urine from aged matched humans. As expected the light chain (25 kDa) of the mouse monoclonal anti-p75NTR used to pull down the p75NTR from was detected after the IP/WB procedure.

p75NTR^ECD^ in human ALS patient urine was also found by mass spectrometry following digestion of immuno-precipitated p75NTR with the enzyme glutamyl endopeptidase. Five peptides from human p75NTR^ECD^ (P08138; Human Tumor necrosis factor receptor superfamily member 16) were identified with 28% sequence coverage of the p75NTR^ECD^ (Table 1; http://www.uniprot.org).

**Table 1:** Identification of p75NTR^ECD^ peptides after Mass Spectrometry of Urine subject to IP.

P08138: 29–250 (p75NTR^ECD^)	Peptide	MH+^A^	Z^B^	Prob	XCorr
70–88 (19)	SVTFSDVVSATEPCKPCTE	2113.9451	2	15.66	3.91
89–101 (13)	CVGLQSMSAPCVE	1437.6195	2	37.73	2.87
102–117 (16)	ADDAVCRCAYGYYQDE	1955.7579	2	63.95	3.74
158–171 (14)	ANHVDPCLPCTVCE	1671.6959	2	44.22	3.34
158–174 (17)	ANHVDPCLPCTVCEDTE	2016.8135	2	61.58	3.49

The sequence and amino acid numbers for human p75NTR (PO8138) are listed for peptides identified. The probability (Prob) and cross correlation score (XCorr) are included as reported by Proteome Discoverer 1.3 (Thermo). MH+^A^ and Z^B^ refer to the average mass of the mono-protonated peptide and the charge state of the peptide respectively.

**Figure 2:**
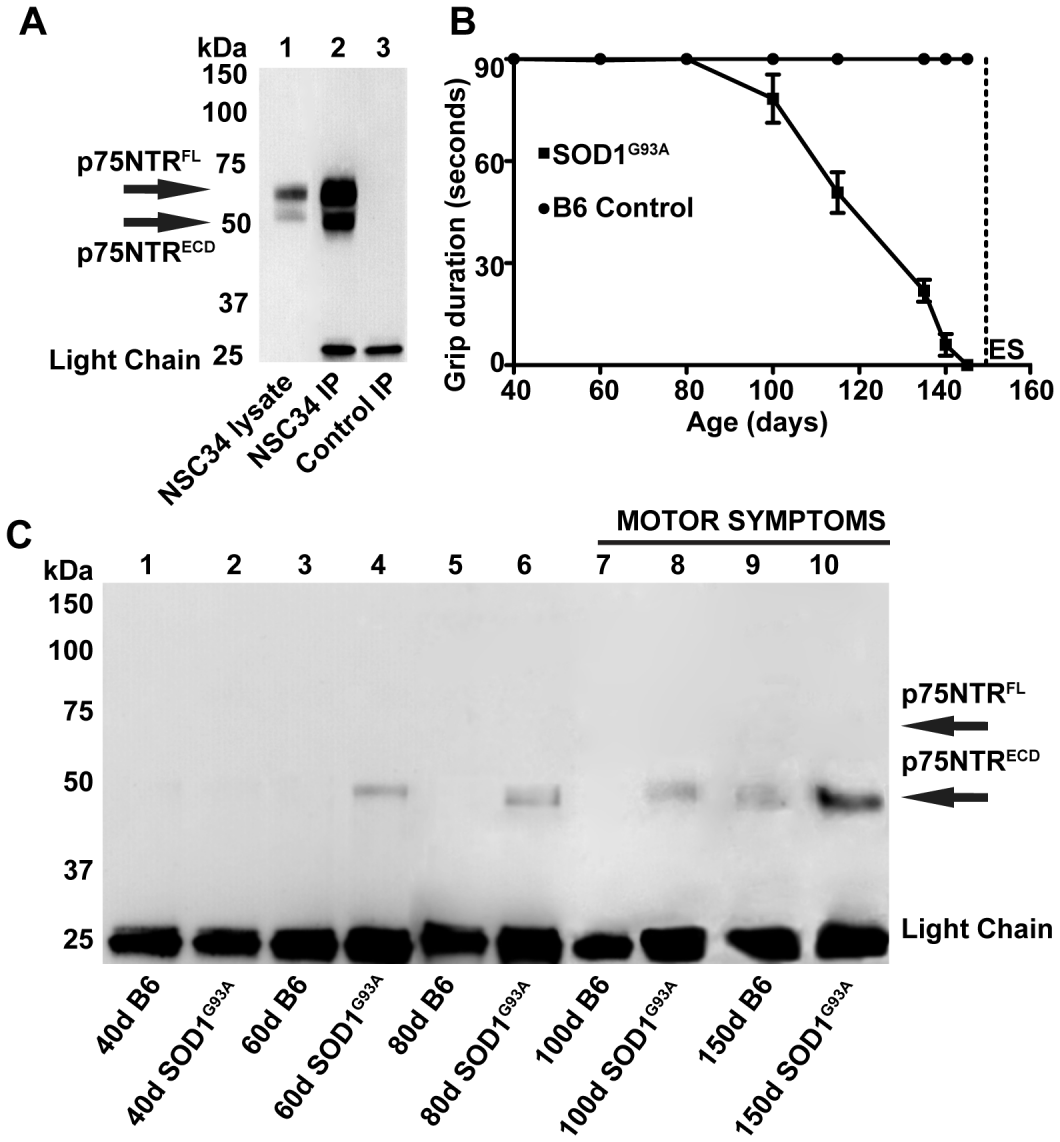
p75NTR^ECD^ was identified by IP/WB of urine from SOD1^G93A^ mice before motor symptoms were detected. **A**. Mouse p75NTR^FL^ and ^ECD^ from motor neuron-like NSC34 cells (5 µg, lane 1) was enriched by the IP/WB (500 µg, lane 2) and not detected in control IP where p75NTR^ECD^ is not present (BSR, 500 µg. lane 3). **B**. Symptoms of amyotrophic lateral sclerosis disease were found in SOD1^G93A^ mice from 100 days by the hanging wire grip duration test (n = 10) in comparison to none in C57BL/6J (B6) control mice (n = 10). End stage (ES) was at 150 days for SOD1^G93A^ mice. **C**. p75NTR^ECD^ was detected after IP/WB of urinary protein from 60d (lane 4) and 80d (lane 6) pre-symptomatic SOD1^G93A^ mice and also 100d (lane 8) and end stage (150d, lane 10). No p75NTR is seen in age matched C57BL/6J control mice until end stage (150d, lane 9). 110 µg of urinary protein was used for each IP of mouse urine and all IP/WB were repeated 3 times with similar results. The light chain of IgG (25 kDa band) was detected after WB of samples subject to IP.

The association of urinary p75NTR^ECD^ with ALS was confirmed in the SOD1^G93A^ mouse model of ALS. The IP/WB procedure detected mouse p75NTR (Fig. 2A). p75NTR^FL^ (∼65 kDA) and p75NTR^ECD^ (∼50 kDA) from mouse NSC34 cells (lane 1) was enriched by IP/WB (lane 2) but not from cells lacking p75NTR (lane 3). In agreement with previous research [24], motor symptoms detected by the hanging wire, grip duration test first appear in SOD1^G93A^ mice at around 100 days (Fig. 2B). However, p75NTR^ECD^ was detected in urine of SOD1^G93A^ ALS mice at 60d (Fig. 2C lane 4), 40 days before clinical symptoms appear. p75NTR^ECD^ was not detected in aged-matched control C57BL/6J (B6) mice (lane 3). p75NTR^ECD^ was also detected at 80d (lane 6) and 100d (lane 8) in SOD1^G93A^ mice but not aged matched C57BL/6J mice (lane 5 and 7). There was a larger amount of p75NTR immuno-precipitated in end-stage SOD1^G93A^ mouse urine (lane 10) than at earlier ages. A low level of p75NTR^ECD^ was detected in 150d-old C57BL/6J mice (lane 9).

### p75NTR^ECD^ measurement in ALS Patients and SOD1G93A mice

Urine samples from 28 patients with sporadic ALS were used in this pilot study (Table 2). The male to female ratio was 17∶11 and 16 patients had limb onset, the rest bulbar. The median disease duration from symptom onset to collection was 20.4 months and 7.8 months from definitive diagnosis by El Escorial criteria [19]. At the time of diagnosis, the median diagnostic delay was 10.8 months (range, 2.6–33.4 months; Table 2). Urine was also collected from controls (n = 12) and those with other neurological disease (Parkinsons' disease, PD: n = 10, Multiple Sclerosis, MS: n = 9). The average age at collection was 67.1 for ALS patients, 59.6 for controls and 58.5 for other neurological disease (69.6 PD and 47.5 for MS).

**Table 2:** Characteristics of Individual ALS Patients and healthy Controls Included in the study.

Characteristics	ALS	Controls
Mean Age (range; yr)	67.1; 44–82	59.6; 40–71
Sex Ratio (M/F)	17/11	6/6
Limb/Bulbar Onset	16/12	NA
Median Diagnostic Delay (time between diagnosis and symptoms; range; mo)	10.8; 2.6–33.4	NA
Median Disease Duration from Symptoms At p75NTR ^ECD^ ELISA (range; mo)	20.4; 4.6–48.6	NA
Median Disease Duration from Diagnosis At p75NTR^ECD^ ELISA (range; mo)	7.8; 0.2–25.1	NA
Riluzole Treatment (Percentage)	70.8%	NA

A sandwich ELISA was developed to measure human and mouse p75NTR^ECD^. A signal to noise ratio of 10 was achieved for 1000 pg/ml human p75NTR^ECD^ (Figure S2A) and 20 for 1000 pg/ml mouse p75NTR^ECD^ (data not shown). The intra-assay and inter-assay co-efficient of variation for measuring p75NTR^ECD^ from the same human urine samples over 16 months using one plate per month was 9.8% and 6.1% respectively. The amount of urine per well that can be sampled was 20% of the total 100 µl (Figure S2B). Towards the end of this study, a commercial ELISA for human p75NTR became available (R&D systems) which was used to re-assay a number of the urine samples. All gave similar results (within 10%) to our in-house assay (Figure S3A and S3B).

**Figure 3:**
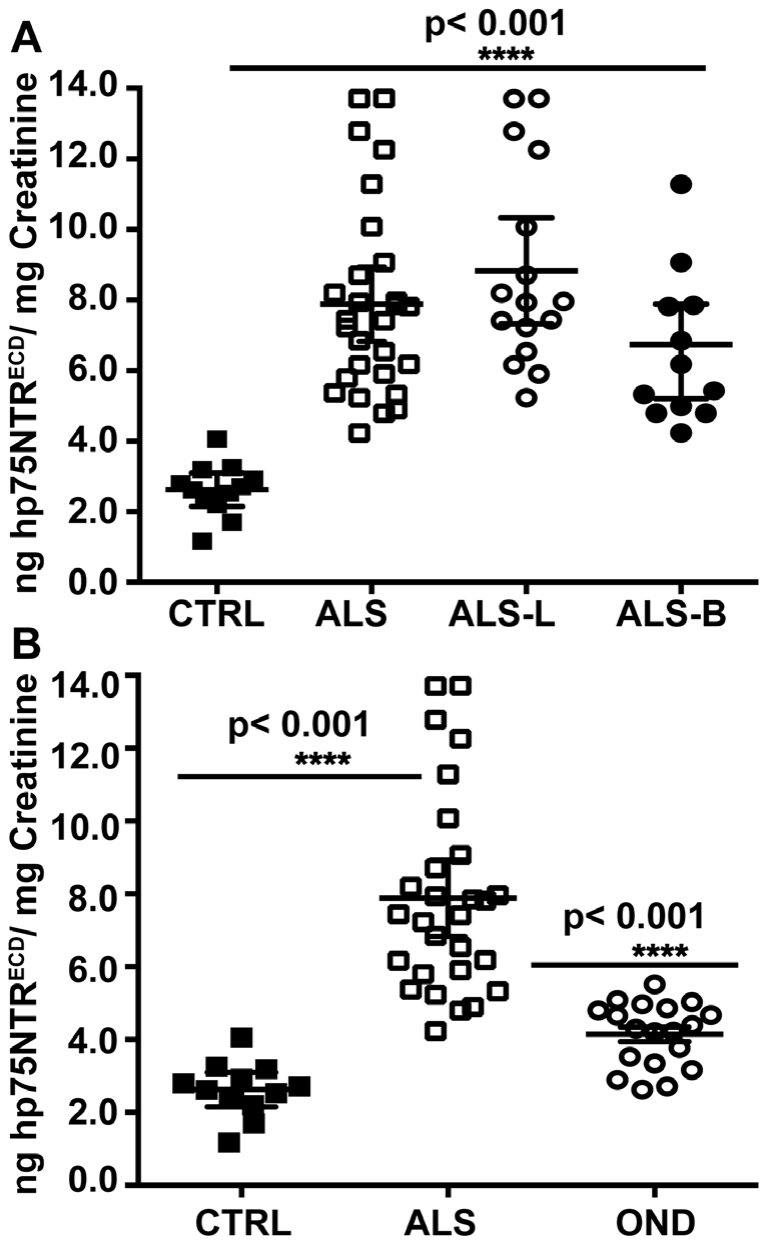
There are significant amounts of p75NTR^ECD^ in the urine of ALS patients compared to healthy controls or people with other neurological diseases. A. p75NTR^ECD^ levels measured by ELISA were significantly higher in all ALS patients (****; p<0.001, n = 28), including limb (ALS-L; n = 16) and bulbar (ALS-B; n = 12) onset in comparison to healthy controls (n = 12). ALS patients with limb onset disease trended to higher mean levels of p75NTR^ECD^ than bulbar onset but this was not significant. B. Levels of p75NTR^ECD^ detected in patients with other neurological conditions (OND; n = 19) are not significantly different to that seen in healthy individuals and are significantly lower (***p<0.001, n = 12) than that seen in ALS patients. All samples were assayed four times in quadruplicate. Levels of urinary p75NTR^ECD^ were standardised to urinary creatinine and data was analysed with one-way ANOVA and Bonferroni's multiple comparison post-hoc test, with significance at alpha  = 0.01 (1% level).

Urinary p75NTR^ECD^ of people living with ALS (7.9±0.5 ng/mg creatinine, n = 28) was significantly higher (p<0.001) than controls (2.6±0.2 ng/mg creatinine, n = 12; Fig. 3A). When ALS patients were classified on site of onset, both limb (8.8±0.7 ng/mg creatinine; n = 16) and bulbar (6.5±0.6 ng/mg creatinine; n = 12) onset were still significantly higher than controls. Those with limb onset had higher mean levels than bulbar onset but this difference was not significant at the 1% level. There was no correlation between urinary p75NTR^ECD^ levels and gender or age (Spearman rank test, Prism 6). ALS patients had a higher mean value of urinary p75NTR^ECD^ than people living with other neurological conditions (7.9±0.5 versus 4.1±0.2 ng/mg creatinine; OND: PD and MS, n = 19), significant at the 1% level (alpha = 0.01, Fig. 3B). In addition, people living with OND had a higher mean urinary p75NTR^ECD^ then controls but this was not statistically significant at the 1% level (4.1±0.2 versus 2.6±0.2 alpha = 0.01, Fig. 3B).

**Figure 4:**
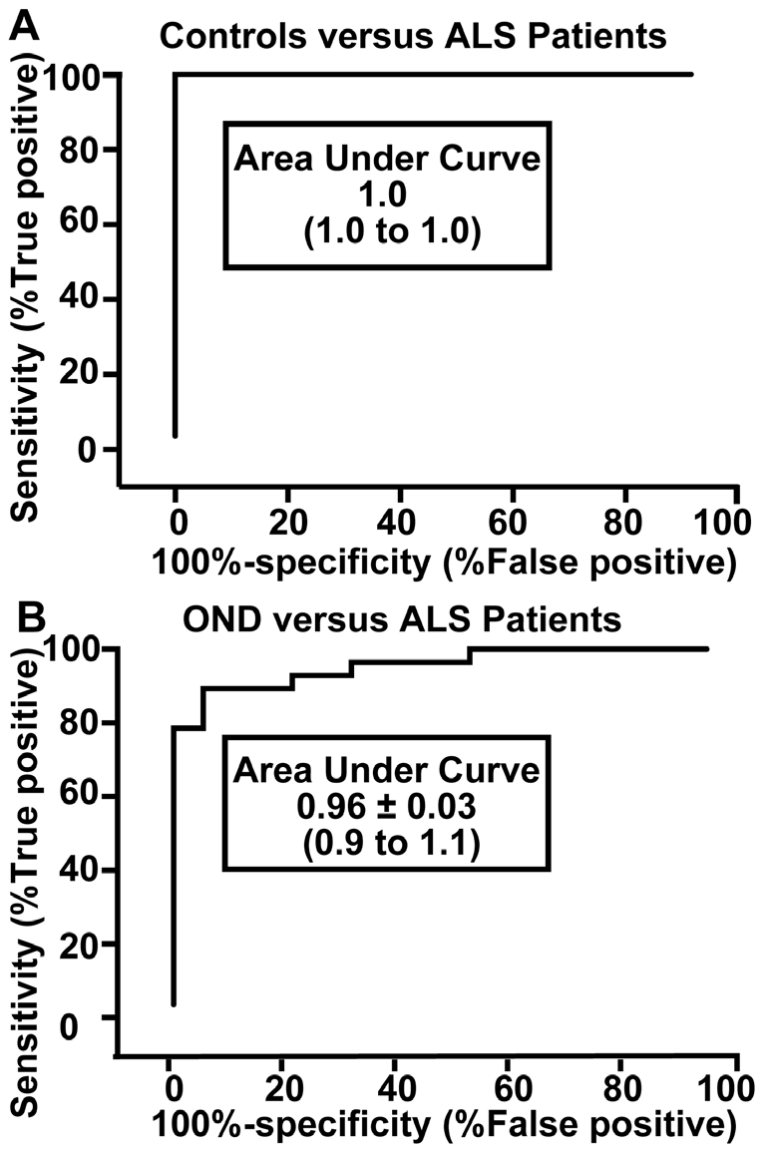
Receiver Operating Characteristic curves for distinguishing ALS patients from A. healthy controls and B. patients with other neurological conditions (OND) using p75NTR^ECD^. A The area under the curve (AUC) indicated ALS patients are distinguished from controls 100% of time (AUC: 1.0) with a 95% confidence interval of 1. B. In comparison, ALS was distinguished from OND (Parkinson's and Multiple Sclerosis) 96% of time, with a 95% confidence interval of 0.9 to 1.1. The cut-off value for distinguishing ALS patients from healthy controls, using the Youden index (Baker and Krame, 2007) was >4.8 and from OND >4.8 (ng p75NTR^ECD^/mg creatinine).

To confirm p75NTR^ECD^ provides utility to distinguish ALS from controls we performed ROC curve analyses of controls versus ALS patients and other neurological diseases (OND). Urinary p75NTR^ECD^ levels can diagnose ALS from healthy controls with 100% specificity and 93% sensitivity (Fig 4A). The area under the curve (AUC) indicated ALS patients are distinguished from controls 100% of time (AUC: 1.0). In comparison, ALS can be distinguished from other neurological disease (PD and MS) with 79% specificity and 93% sensitivity (Fig 4B). ALS patients are distinguished from other neurological disease 96% of the time (AUC: 0.96) with a 95% confidence interval of 0.9 to 1.1 (Fig. 4B). The cut-off value for distinguishing ALS patients from healthy controls, using the Youden index [29] was >4.8 and from OND >4.8 (ng p75NTR^ECD^/mg creatinine).

### Association of p75NTR^ECD^ with progression of ALS

**Figure 5:**
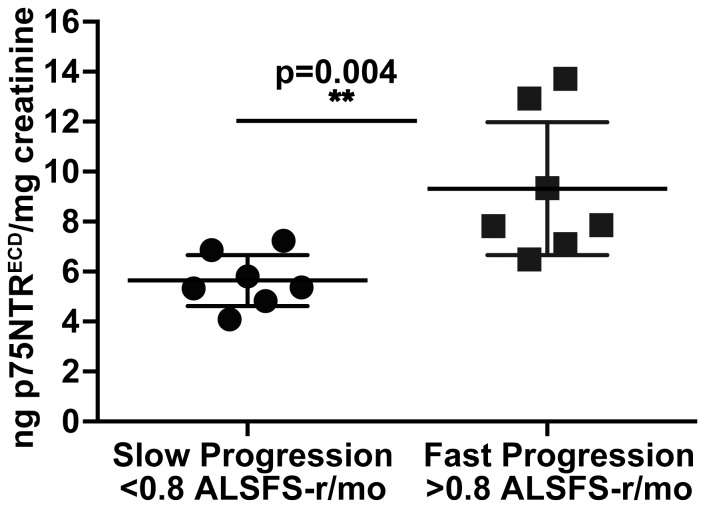
There are significant amounts of p75NTR^ECD^ in the urine of rapidly progressing compared to slowly progressing ALS patients. Scatter plots show urinary p75NTR^ECD^ concentrations in ‘slowly progressive ALS’ (n = 7) and ‘rapidly progressive ALS’ (n = 7). The horizontal lines represent the mean, with 95% confidence interval. Progression rate is decline in ALSFRS-r per month. *Mann–Whitney test (p = 0.004).

A pilot project was undertaken where 14 ALS patients gave samples of urine on consecutive 3- month intervals with clinical data including ALSFRS-r also recorded. These 14 patients had a mean age of 65, included 9 males, 7 females, with equal distribution of limb and bulbar onset ALS (Table 3). Urinary p75NTR^ECD^ levels were measured 3-monthly, and decline in ALSFRS-r per month, recorded. Seven patients were defined as ‘rapidly progressive’ based on ALSFRS-r disease progression rate (>0.8 ALFRS-r/month) and 7 as ‘slowly progressive’ (<0.8 ALSFRS-r/month), as previously indicated [9]. Rapidly progressing patients had significantly higher urinary p75NTR^ECD^ levels compared to slowly progressive patients (Mann–Whitney test, p = 0.0041; Fig. 5). p75NTR^ECD^ in SOD1^G93A^ mice urine was also measured by ELISA over disease progression. There was an increase in p75NTR^ECD^ levels from 38±2.1 ng p75NTR^ECD^/mg creatinine pre-symptomatically (60 days) to 78.3±7.8 ng p75NTR^ECD^/mg creatinine at end stage (n = 6). Healthy C57BL/6J mice aged-matched to SOD1^G93A^ mice had small but detectable levels of p75NTR^ECD^ with no correlation to age. For example, 150 day-old C57BL/6J aged-matched to end-stage SOD1^G93A^ mice had 17.2±1 ng p75NTR^ECD^/mg (n = 6), confirming the earlier observation in Fig 2.

**Table 3:** Characteristics of Patients in ALS Progression Study.

Characteristics	ALS Patient Data
Mean Age (range; year)	65.0; 44–83
Sex Ratio (M/F)	9/5
Limb/Bulbar onset	7/7
Median Diagnostic Delay (range; mo)	5.2; 2.6–35.3
ALSFRS-r; median (range)	38.0; 29–45

## Discussion

To our knowledge this is the first time that urinary p75NTR^ECD^ has been proposed as a candidate marker of ALS/MND. p75NTR mediates dual opposing functions of cell survival and death that is controlled by the presence or absence of neurotrophins and includes apoptosis in the adult nervous system upon injury and degeneration [31]. In our pilot study of 28 ALS patients and 12 controls we have definitively identified p75NTR^ECD^ in human urine of ALS patients and shown the mean levels are near to 3 fold higher than healthy controls (7.8±0.5 versus 2.6±0.3 ng/mg creatinine). In addition, we found higher urinary p75NTR^ECD^ levels in patients with rapidly progressive disease, as measured by ALSFRS-r progression rate, a validated marker for predicting progression in ALS [32].

Our finding of p75NTR^ECD^ in the urine of ALS patients is supported by IP/WB and mass spectrometry. There is only one previous report of a urinary biomarker for ALS, Collagen alpha-4(IV), [33], and this work was not progressed further. The less complex proteome of urine makes it simpler to search for biomarkers then, for example serum [34]. The low amount of p75NTR^ECD^ we measured in urine of healthy controls agrees with work indicating p75NTR^ECD^ corrected for creatinine is low but detectable in adult urine even at old age [14]. The p75NTR^ECD^ found in urine of ALS patients is not the result of bladder dysfunction as the motor neurons controlling bladder function are spared in ALS [35] and urinary problems not generally reported. Measurements of human urinary p75NTR^ECD^ by ELISA were robust with low intra-assay and inter-assay variability (>10%) when urine stored from 0–16 months was tested. This can be contrasted with serum where a more complex proteome may hinder reliable ELISAs [8], [34].

We also identified p75NTR^ECD^ in urine from SOD1^G93A^ mice. This was not surprising as previous studies have revealed p75NTR is re-expressed in motor neurons of SOD1^G93A^ mice and absent in aged matched controls [17], [36]. In addition, p75NTR^ECD^ appears in rat urine following sciatic nerve injury [15] that may derive from associated Schwann cells [15], [37]. Subsequent research has revealed p75NTR re-expression after nerve injury may be part of a homeostatic program that removes defective neurons, axons and synapses upon injury and degeneration [31]. It is highly unlikely any non-neuronal tissue would re-express p75NTR and release p75NTR^ECD^ as the site of ‘injury’ in ALS is neuronal. The importance of p75NTR in motor neuron degeneration is indicated by improvement in survival and regeneration of axotomized motor neurons in p75NTR knockout mice compared to control animals [12]. Since p75NTR is up-regulated in Schwann cells in ALS patients post mortem [38], it is reasonable to conclude that p75NTR^ECD^ in urine of ALS mice and patients is derived not only from motor neurons but also from Schwann cells.

Urinary p75NTR^ECD^ was not observed in control C57BL/6J mice by IP/WB until they were 150 days of age old. However, the intensity of protein bands was much lower than for aged-matched SOD1^G93A^ mice. ELISAs that are more sensitive at detecting p75NTR^ECD^ than IP/WB, indicated normal adult mouse urine throughout life contains detectable p75NTR^ECD^, but the levels were significantly lower than SOD1^G93A^ mice. It is not surprising that small amounts of p75NTR^ECD^ were detected in normal adult mice urine since a previous report showed p75NTR^ECD^ is at low but detectable levels in adult human urine [14]. We also found no correlation between urinary p75NTR^ECD^ levels and age for healthy human controls, which agrees with a previous study [14]. Therefore minor amounts of urinary p75NTR^ECD^ may be present from adulthood, the source of which is unknown, but this does not confound our finding that p75NTR^ECD^ levels in ALS patients and SOD1^G93A^ mice are significantly higher than controls.

There are no published studies on urinary p75NTR in MS or PD. While we found the mean urinary p75NTR^ECD^ levels in other neurological disease (MS and PD) was slightly higher than healthy controls, this was not statistically significant. Although larger numbers are required to confirm the validity of our observations, our pilot study used conservative tests of significance. Previous studies have indicated p75NTR is up-regulated in oligodendrocytes of active brain plaques from MS patients [39] and up-regulated in some basal forebrain cholinergic neurons in PD [40]. However, these are more limited sources of p75NTR in comparison to ALS. Given that the source of p75NTR^ECD^ in ALS (motor neuron pool and associated Schwann cells) is potentially larger than for MS and PD our results are consistent with the underlying physiology and pathology.

The only published investigation of urinary p75NTR in humans [41] found there was a significant elevation in p75NTR^ECD^ in human urine of mildly demented people compared to controls. They reported ∼2.0 µg of p75NTR^ECD^/ml urine for mildly demented people and 1.1 µg of p75NTR^ECD^/ml urine healthy controls [41] indicating p75NTR^ECD^ comprises ∼5.0% of normal urinary protein. In comparison, we show 0.0075 µg p75NTR^ECD^/ml urine and 0.002 µg p75NTR^ECD^/ml urine for ALS patients and healthy controls, respectively. This constitutes ∼0.02% of normal urinary protein. Lindner and colleagues [41] acknowledged their urine storage may have affected p75NTR^ECD^ measurements. In contrast, our study used the Kidney and Urine Proteome Project standards [22], including collection within 3 h, centrifugation and storage at −80°C. In addition, our intra-assay co-efficient of variation below 10% for measurements obtained over 16-months indicates urine storage is not a factor. Given that Lindner's study was performed 20 years ago [41] and has never been confirmed, it is important to re-evaluate urinary p75NTR^ECD^ in people living with dementia. Since dementia occurs at a more advanced age than ALS (>70′s; [42] compared to ALS (50′s; [19])) and we found no correlation of p75NTR^ECD^ levels with age, dementia may not be relevant to using p75NTR^ECD^ as a biomarker. However, further analysis may be required to measure p75NTR in other neurological diseases where differential diagnosis from ALS can be difficult [43]. These include neurologic diseases that closely resemble ALS, such as primary lateral sclerosis, or ALS “mimic diseases” such as post-poliomyelitis syndrome, multifocal motor neuropathy and endocrinopathies, especially hyper parathyroid or hyperthyroid states [19], [43].

Our pilot data indicates the usefulness of urinary p75NTR^ECD^ as a biomarker to distinguish ALS patients from healthy controls and other neurological disease (MS or PD). A value of greater than 4.8 ng p75NTR^ECD^/mg creatinine distinguished ALS from healthy controls with 93% sensitivity and 100% specificity. This same cut-off value distinguished ALS from other neurological disease with 93% sensitivity and 79% specificity. The sensitivity and specificity of p75NTR^ECD^ compared to healthy controls is higher than pNF-H in the serum [44], [45] and the CSF [8], [46], [47]. A comparison with CSF derived NF-L is difficult to make as no healthy controls were used in the study [9]. p75NTR^ECD^ has higher sensitivity and specificity than neuro-imaging markers such as MRI [48]. However, single biomarkers are unlikely to meet all the criteria needed to accurately diagnose ALS [2], [5]. For example, pNF-H in combination with complement C3 values in CSF has improved specificity and sensitivity compared to pNF-H alone [8]. Therefore urinary p75NTR^ECD^ could be added to other probable diagnostic markers such as pNF-H/C3 from CSF and tested in larger cohorts.

Indicators of ALS progression are needed especially in clinical trials of potential disease modifying agents. This need has been highlighted by the ‘Volcano Group’ that has also suggested ways to facilitate the search for progression markers that can be translated to clinical trials for ALS [2]. Our pilot data found higher urinary levels of p75NTR^ECD^ in patients with rapidly progressive disease, as measured by the progression rate. Previous studies reported high levels of pNF-H and NF-L in the CSF of patients with ALS and faster progression of the disease [46], [9]. The high p75NTR^ECD^ levels in rapidly progressive patients may reflect the intensity of the neurodegenerative process and may point towards a rapid progression of the disease also in its early phase. Our data also indicates p75NTR^ECD^ appears to increase over the progression of disease in SOD1^G93A^ mice, further highlighting p75NTR^ECD^ may be a marker of disease progression.

In conclusion, we found that urinary p75NTR^ECD^ levels are increased in ALS and correlate with disease progression. Although this is a small study on a select sample of patients, these findings indicate p75NTR^ECD^ may be a candidate marker of disease progression. Further work should include measurement of p75NTR^ECD^ in other mimic neurodegenerative disorders. Considering ALS is a heterogeneous disease and that p75NTR^ECD^ marks only the process of motor neuron degeneration and Schwann cell activity, a combined use of p75NTR^ECD^ with other candidate markers in larger sample populations could be examined.

## Supporting Information

Figure S1 Human p75NTR extracellular domain (p75NTR^ECD^) and full-length p75NTR (p75NTR^FL^) was detected by immuno-precipitation/western blot (IP/WB), using MLR2 as pull-down and rabbit anti-p75NTR^ECD^ (Alomone labs) as detection. Human p75NTR^ECD^ (50–55 kDA) and p75NTR^FL^ (70–75 kDA) from A875 melanoma cells (5 µg, lane 3) was enriched after IP of 500 µg of cell lysate (lane 2). No p75NRTR was detected after IP of control cells lacking p75NTR (BSR, 500 µg, lane 3). The light chain of IgG (25 kDa band) was detected after western blot (WB) of samples subject to IP.
(DOC)

Figure S2 Standard curve indicates sensitivity of enzyme linked immunosorbent assay (ELISA) for human urinary p75NTR^ECD^. **A**. Representative standard curve (diamond symbol; n = 12 with standard deviation) shows the assay is linear up to 1000 pg/ml of human p75NTR^ECD^ with a signal to noise ratio (S/N) of 10. **B**. Linearity of the ELISA as a function of urine volume. Urine from an ALS patient (round symbols) and healthy individual (square symbols) was subject to p75NTR^ECD^ ELISA, with the results being linear up to 20 µl of urine per 100 µl assay. The curve is from 6 separate assays in triplicate, with standard deviation. Goodness of fit to straight lines (r^2^) was determined in Prism6.
(DOC)

Figure S3 In-house and commercial enzyme linked immunosorbent assay (ELISA) for urinary p75NTR^ECD^ produces similar measurement. **A**. Representative standard curve for In-House ELISA (circle symbol; n = 4 with standard deviation) compared to commercial R&D kit (square symbol; n = 4 with standard deviation) shows both assays are linear from 50 to 1000 pg/ml of human p75NTR^ECD^. Standard deviation and goodness of fit to straight lines (r^2^) was determined in Prism6. **B**. There was no significant difference (p = 0.97) between levels of urinary p75NTR^ECD^ detected by either the In-house or commercial ELISA in ALS patient or healthy controls urine (n = 4 with standard deviation error bars). Significance was tested by an unpaired t-test using Prism6.
(DOC)
