# Supplementary figures and images for: The Extracellular Domain of Neurotrophin Receptor p75 as a Candidate Biomarker for Amyotrophic Lateral Sclerosis
Source: PLoS One. 2014 Jan 27;9(1):e87398. doi: 10.1371/journal.pone.0087398 (PMC3903651; doi:10.1371/journal.pone.0087398)

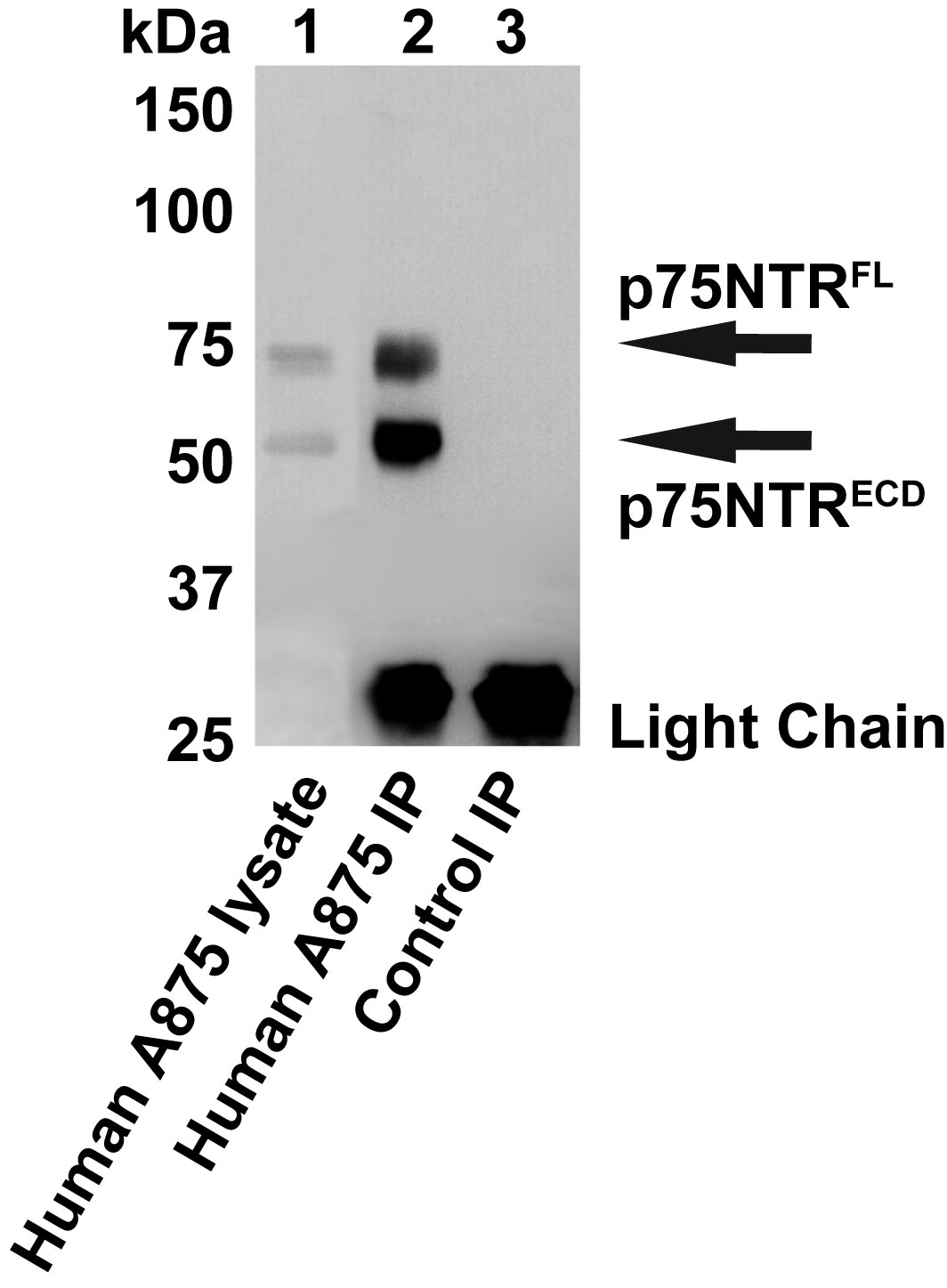

Supplement: Figure S1 — Human p75NTR extracellular domain (p75NTRECD) and full-length p75NTR (p75NTRFL) was detected by immuno-precipitation/western blot (IP/WB), using MLR2 as pull-down and rabbit anti-p75NTRECD (Alomone labs) as detection. Human p75NTRECD (50–55 kDA) and p75NTRFL (70–75 kDA) from A875 melanoma cells (5 µg, lane 3) was enriched after IP of 500 µg of cell lysate (lane 2). No p75NRTR was detected after IP of control cells lacking p75NTR (BSR, 500 µg, lane 3). The light chain of IgG (25 kDa band) was detected after western blot (WB) of samples subject to IP. (DOC) [file pone.0087398.s001.doc]

**
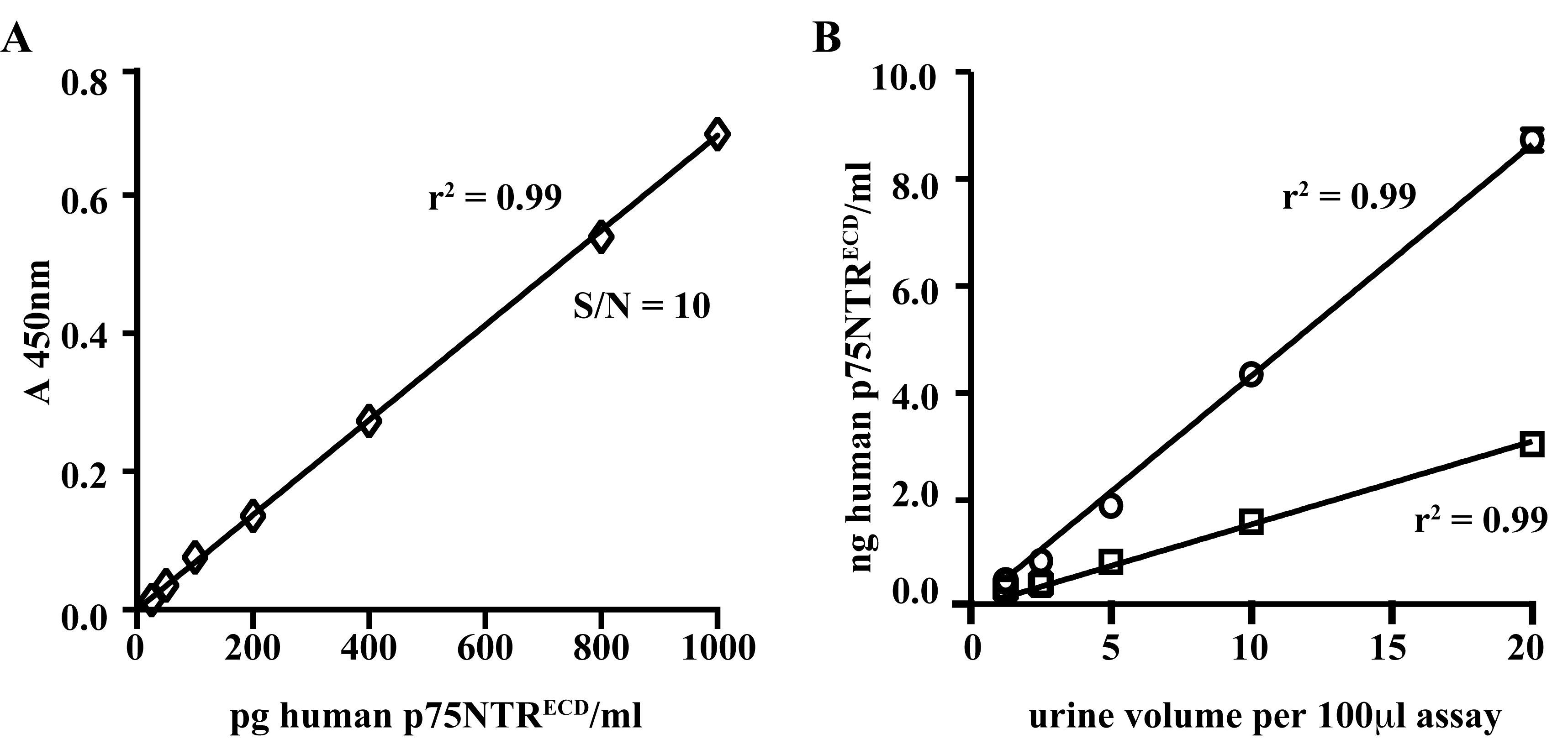
**

Supplement: Figure S2 — Standard curve indicates sensitivity of enzyme linked immunosorbent assay (ELISA) for human urinary p75NTRECD. A. Representative standard curve (diamond symbol; n = 12 with standard deviation) shows the assay is linear up to 1000 pg/ml of human p75NTRECD with a signal to noise ratio (S/N) of 10. B. Linearity of the ELISA as a function of urine volume. Urine from an ALS patient (round symbols) and healthy individual (square symbols) was subject to p75NTRECD ELISA, with the results being linear up to 20 µl of urine per 100 µl assay. The curve is from 6 separate assays in triplicate, with standard deviation. Goodness of fit to straight lines (r2) was determined in Prism6. (DOC) [file pone.0087398.s002.doc]

**
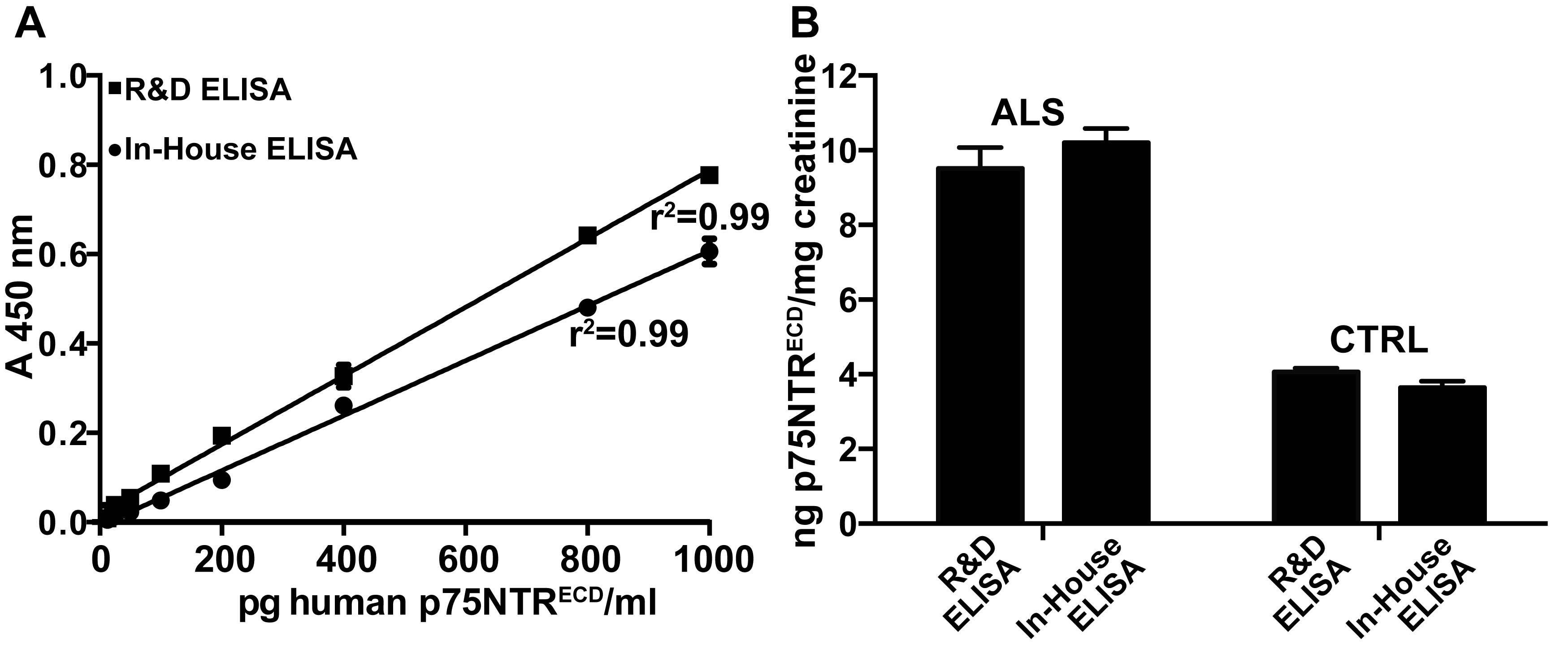
**

Supplement: Figure S3 — In-house and commercial enzyme linked immunosorbent assay (ELISA) for urinary p75NTRECD produces similar measurement. A. Representative standard curve for In-House ELISA (circle symbol; n = 4 with standard deviation) compared to commercial R&D kit (square symbol; n = 4 with standard deviation) shows both assays are linear from 50 to 1000 pg/ml of human p75NTRECD. Standard deviation and goodness of fit to straight lines (r2) was determined in Prism6. B. There was no significant difference (p = 0.97) between levels of urinary p75NTRECD detected by either the In-house or commercial ELISA in ALS patient or healthy controls urine (n = 4 with standard deviation error bars). Significance was tested by an unpaired t-test using Prism6. (DOC) [file pone.0087398.s003.doc]
